# Gait Patterns as Digital Markers of Post‐Stroke Cognitive Impairment: A Comparative Analysis of Real‐World and Laboratory Assessments

**DOI:** 10.1002/alz70858_106287

**Published:** 2025-12-26

**Authors:** Shimaa Aboudeif, Britney Denroche, Bhavana Gill, Armin Ghayur Sadigh, Philip A Barber, Sayeh Bayat

**Affiliations:** ^1^ University of Calgary, Calgary, AB, Canada; ^2^ University of Calgary, Cumming School of Medicine, Calgary, AB, Canada; ^3^ Hotchkiss Brain Institute, Calgary, AB, Canada; ^4^ KITE ‐ Toronto Rehabilitation Institute, University Health Network, Toronto, ON, Canada; ^5^ University of Toronto, Toronto, ON, Canada

## Abstract

**Background:**

Transient ischemic attack (TIA), or ministroke, is a major risk factor for dementia, with post‐TIA dementia risks reaching 20% at 5 years, 30% at 15 years, and 48% at 25 years. Given this high risk, health authorities emphasize the need for prediction and early detection. Subtle gait changes, emerging as early cognitive decline markers, are often assessed in laboratory settings. However, leveraging passively collected data from wearable technology enables continuous, real‐world monitoring of mobility and cognitive health, offering an ecologically valid approach for early dementia detection.

**Objective:**

To compare gait kinematic parameters between healthy individuals and TIA patients and assess the similarities in real‐world and laboratory settings.

**Method:**

Gait data were collected from three TIA patients (67.67±2.52 years) and four healthy controls (67.75±4.19 years) using lumbar‐worn wearable sensors. Real‐world gait monitoring spanned 21 days; laboratory‐based assessments included the Timed Up and Go (TUG) test and dual‐task gait test. Key gait parameters were analyzed including step length, gait speed, stride length and cadence. Statistical comparisons were performed using independent t‐tests (*p* < 0.05).

**Result:**

In real‐world settings, TIA patients showed significantly larger vertical axis stride‐step regularity difference (‐2.71±0.07 vs. ‐2.57±0.03, *p* = 0.03), step length (0.91±0.02 m vs. 0.66±0.05 m, *p* = 0. 0.0009), stride length (1.82±0.04 m vs. 1.33±0.1 m, *p* = 0.001), gait speed (1.71±0.05 m/s vs. 1.19±0.08 m/s, *p* = 0.0004), and cadence (115.22±3.65 vs. 107.06±2.36, *p* = 0.029) compared to healthy controls. Between the real‐world and laboratory settings in TIA patients, the absolute difference between stride and step regularity for the vertical axis with significantly larger in real life than in the lab (0.06±0.01 vs. 0.03±0.01, *p* = 0.014). While there was no significant difference between the two settings in healthy controls.

**Conclusion:**

Preliminary results suggest that TIA significantly affects some gait parameters, particularly in real‐world settings. These findings demonstrate that real‐world gait measurements may serve as sensitive and scalable markers of mobility decline, highlighting the potential of wearable technology for early detection and continuous monitoring of TIA. However, larger studies are needed to validate these findings due to the current small sample size.

